# Novel prognostic features and personalized treatment strategies for mitochondria-related genes in glioma patients

**DOI:** 10.3389/fendo.2023.1172182

**Published:** 2023-04-05

**Authors:** Ji Wu, Jiabin Zhou, Yibo Chai, Chengjian Qin, Yuankun Cai, Dongyuan Xu, Yu Lei, Zhimin Mei, Muhua Li, Lei Shen, Guoxing Fang, Zhaojian Yang, Songshan Cai, Nanxiang Xiong

**Affiliations:** ^1^ Department of Neurosurgery, Zhongnan Hospital, Wuhan University, Wuhan, China; ^2^ Department of Neurosurgery, Affiliated Hospital of Youjiang Medical University for Nationalities, Baise, China; ^3^ Department of Neurosurgery, Red Cross Hospital of Yulin City, Yulin, China

**Keywords:** candidate drugs, glioma, mitochondria, prognostic, signature

## Abstract

**Background:**

Gliomas are the most common intracranial nervous system tumours that are highly malignant and aggressive, and mitochondria are an important marker of metabolic reprogramming of tumour cells, the prognosis of which cannot be accurately predicted by current histopathology. Therefore, Identify a mitochondrial gene with immune-related features that could be used to predict the prognosis of glioma patients.

**Methods:**

Gliomas data were downloaded from the TCGA database and mitochondrial-associated genes were obtained from the MITOCARTA 3.0 dataset. The CGGA, kamoun and gravendeel databases were used as external datasets. LASSO(Least absolute shrinkage and selection operator) regression was applied to identify prognostic features, and area and nomograms under the ROC(Receiver Operating Characteristic) curve were used to assess the robustness of the model. Single sample genomic enrichment analysis (ssGSEA) was employed to explore the relationship between model genes and immune infiltration, and drug sensitivity was used to identify targeting drugs. Cellular studies were then performed to demonstrate drug killing against tumours.

**Results:**

COX assembly mitochondrial protein homolog (*CMC1*), Cytochrome c oxidase protein 20 homolog (*COX20*) and Cytochrome b-c1 complex subunit 7 (*UQCRB*) were identified as prognostic key genes in glioma, with *UQCRB*, *CMC1* progressively increasing and *COX20* progressively decreasing with decreasing risk scores. ROC curve analysis of the TCGA training set model yielded AUC (Area Under The Curve) values >0.8 for 1-, 2- and 3-year survival, and the model was associated with both CD8+ T cells and immune checkpoints. Finally, using cellMiner database and molecular docking, it was confirmed that *UQCRB* binds covalently to Amonafide *via* lysine at position 78 and threonine at position 82, while cellular assays showed that Amonafide inhibits glioma migration and invasion.

**Conclusion:**

Our three mitochondrial genomic composition-related features accurately predict Survival in glioma patients, and we also provide glioma chemotherapeutic agents that may be mitochondria-related targets.

## Introduction

Gliomas are the most prevalent type of tumor found in the brain and spinal cord, with an estimated global incidence of 6 cases per 100,000 people annually (1). These tumors are classified into four grades, ranging from Grade I to Grade IV, with Grade II, III, and IV gliomas having progressively lower median overall survival rates (2, 3). Low-grade gliomas have a better prognosis than glioblastomas, with 70% of patients developing a glioblastoma within 10 years; however, overall, patients with gliomas have a poor prognosis and shorter survival (4). Early diagnosis and risk assessment are essential for the successful treatment of gliomas, and neuroimaging techniques such as magnetic resonance imaging (MRI) are commonly used for diagnosis and monitoring. However, imaging may not be able to differentiate between true tumor progression and pseudo-progression (5), leading to delays in treatment and possible clinical interventions. Current treatments for gliomas include maximal surgical resection of the neoplasm, postoperative radiotherapy, and chemotherapy; however, due to the aggressive nature of gliomas, which are resistant to both radiotherapy and chemotherapy (1), clinical treatment remains challenging. Therefore, the development of effective and innovative prognostic models and relevant drug targets is an urgent and critical task for the early diagnosis and drug treatment of gliomas.

Mitochondria are organelles involved in bioenergetics, biosynthesis, and signaling, and are also components of stress perception, providing a powerful guarantee of cellular adaptation to the environment. Recent evidence suggests that one of the reasons for the formation of treatment-resistant cells in gliomas with mitochondrial involvement is metabolic reprogramming, and that glioma cells frequently metabolize glucose to lactate even in the presence of oxygen (the Warburg effect), allowing tumor cells to use glucose-derived carbon to synthesize essential cellular components while still producing sufficient Adenosine triphosphate (ATP) to power cellular responses (6, 7). Glioma cells are highly reliant on mitochondrial oxidative phosphorylation (OXPHOS) for ATP generation, and glioma cells with a preference for aerobic glycolysis can produce OXPHOS in the absence of glucose (8, 9). This indicates that tumor cells can undergo metabolic reprogramming through the action of mitochondria and possess the ability to undergo different energy metabolic conversions depending on tumor microenvironmental conditions, which facilitates tumor growth. However, the relationship between mitochondria-related genes and the prognosis of glioma patients remains to be elucidated.

In this study, we first determined the expression of Mitochondrial related differentially expressed genes (Mit-DEGs)in glioma samples by obtaining mRNA expression data from the mitochondrial gene set and glioma and corresponding clinical data from patients, respectively, based on publicly available data. We then constructed prognostic models of mitochondria-associated genes in the TCGA dataset and validated them by characterizing the mitochondria-associated genes in the CGGA, kamoun, and gravendeel datasets. Additionally, we used three normal brain tissues and four glioma tissues to examine protein expression in the models, and further annotated the risk groups of the prognostic models for function and explored the mechanisms. Finally, we identified drugs as mitochondria-related therapeutic targets for gliomas and used molecular docking to demonstrate that *UQCRB* binds covalently to Amonafide *via* lysine at position 78 and threonine at position 82, and that cellular assays suggest that Amonafide inhibits migration and invasion of gliomas.

## Materials and methods

### Data download

RNA-seq data for 529 low-grade gliomas (LGG), 168 High grade gliomas (GBM) and 1152 normal brain samples were downloaded from the UCSC database (http://xena.ucsc.edu/)(10) and in the TCGA database (https://www.cancer.gov/about-nci/organization/ccg/research/structural-genomics/tcga) to download RNA-seq data for 693 glioma samples and corresponding patient clinical data, with clinical and RNA-seq data for 693 and 325 glioma samples downloaded from the CGGA database (http://www.cgga.org.cn/). Other glioma data from the GlioVis database (http://gliovis.bioinfo.cnio.es/)(11).were used as from the Mitochondrial Associated Gene Dataset (https://www.broadinstitute.org/mitocarta/mitocarta30-inventory-mammalian-mitochondrial-proteins-and-pathways.) to retrieve a total of 809 mitochondrial genes relevant in brain tissue (12) and the above glioma data include LGG and GBM

### Differentially expressed gene search

Gene expression data from normal brain tissues of ucsc were merged with gene expression data from TCGA glioma, and differentially expressed genes were obtained by screening the tissues for high and low expression using R software (version 4.2.1), with the following screening conditions: (fdrFilter=0.05; logFCfilter=2). The mitochondria-related genes among the obtained brain tissues were intersected with the (TCGA-LGG and TCGA-GBM) differentially expressed genes and considered as differentially expressed mitochondria-related genes in glioma.

### Prognostic model construction and validation

The differentially expressed mitochondrial-associated genes were extracted from the TCGA, CGGA, kamoun and gravendeel databases by R software, and the differentially expressed mitochondrial gene expression matrices were subjected to one-way Cox regression analysis to determine prognosis (p<0.05). One-way Cox regression of prognostic genes was included in the LASSO regression, which was performed by using the R package “glmnet” (13) and the R package “survminer” to compare Overall Survival (OS) between high and low risk groups. The above operations were performed on the CGGA, kamoun, gravendeel training set and validation set.

The availability and calibration curves of the nomogram graphs were evaluated by using R software to create Nomogram prognostic plots TCGA and CGGA cohorts of factors based on the independent prognostic model (14), by c-index of univariate and multivariate Cox regression analysis. Calibration curves were also plotted for 1-, 2- and 3-year predictions, assessing the maintenance of agreement between predictions and actual survival.

### Prognostic model gene set enrichment analysis

GSEA has been widely used for the identification of potential pathways (15). Explore the impact of gliomas and their diagnostic genes on disease progression mechanisms, and the above results were visualized using the limma package.

### Immunological correlation evaluation

As in our previous work, ssGSEA was used (16). In brief. Expression data (ESTIMATE) was used to estimate stromal and immune cells in malignant tumours used to calculate tumour purity, stromal score, immune score and ESTIMATE score in glioma cases. The relationship between immune function and immune cells and prognostic models was explored. Scoring files were merged with clinical data to explore the relationship between subgroups of the prognostic model and the survival of immune cells, and to explore the correlation between key differential genes in the prognostic model and immune cells in GBM versus LGG.

### Exploring the expression and prognosis of key DEGs

These key DEGs were imported onto the Human Protein Atlas (HPA) for validation (http://www.proteinatlas.org/)(17). Three normal tissue and four glioma tissue samples were also used for Western Blot protein content validation.

### Screening for potential small molecule drugs and molecular docking

The CellMiner database (https://discover.nci.nih.gov/cellminer/home.do) was used to find relevant drugs for the model genes (18). and the results were finally visualised using ggplot2. The 2D structures of potential active ingredients and key DEG targets were then downloaded from Pubchem (https://pubchem.ncbi.nlm.nih.gov/) and PDB database (http://www.rcsb.org/) respectively, and then, using autoDOCK software(19), the molecular target to small molecule drugs was completed docking analysis. Finally, the molecular docking of drug small molecules to protein macromolecules was verified using PyMOL software (v.2019.0102) to demonstrate their good interaction sites.

### Cell culture

Human glioma cell line U251、 A172 and Human astrocytes SVGp12 were purchased from American Type Culture Collection (ATCC, USA). U251 and A172 cells were cultured in DMEM/high-glucose medium containing 10% FBS and 1% penicillin−streptomycin and placed in a 5% CO2 humidified incubator at 37°C.

### Reagents

Amonafide (CAS No. 69408-81-7, 10 mM*1 mL in DMSO) was purchased from MedChemExpress (MCE, Shanghai, China) and stored at -20°C. Amonafide was diluted at a concentration of 500 μM as the working solution and stored at -4°C. Dulbecco’s modified Eagle’s medium (DMEM)/high glucose and phosphate-buffered saline (PBS) were purchased from Servicebio (Wuhan, China). Foetal bovine serum (FBS) was purchased from HYCEZMBIO (Wuhan, China). Penicillin−streptomycin was purchased from Gibco (Grand Island, NY, USA). GAPDH antibody (AC001), *CMC1* antibody (A21111), and HRP Goat Anti-Rabbit IgG (AS014) were purchased from ABclonal (Wuhan, China). *UQCRB* antibody (10756-1-AP), *COX20* antibody (25752-1-AP) were purchased from Proteintech Group, Inc (Wuhan, China).

### Cell viability assay

Cells were resuspended and seeded in 96-well plates at a density of 3000 cells per well. After cell adherence, cells were treated with serial concentrations of Amonafide for 24 h and 48 h. According to the protocol of the Cell Counting Kit-8 (Servicebio, Wuhan, China), the absorbance value of each well was measured by using a multifunctional microplate reader (SpectraMax iD3, USA) at 450 nm after adding 10 μl CCK-8 reagent. Cell viability was calculated based on the absorbance values by using the formula: cell viability (%) = (Adrug - Ablank)/(Acontrol- Ablank) × 100%.

### Colony formation assay

A total of 1000 cells per well were plated in a 6-well plate. After cell adherence, the cells were pretreated with Amonafide for 24 h. Then, the cells were cultured with 2 ml medium containing 10% foetal bovine serum in an incubator and refreshed every three days. Fourteen days later, the cells were fixed in 4% paraformaldehyde and stained with crystal violet solution (Servicebio, Wuhan, China). Colonies were captured using a camera and calculated using Image J software.

### Wound healing assay

Cells were resuspended with serum-free medium and plated into 6-well plates at a density of 1×106 cells per well overnight. Next day, a 200-μl sterile pipette tip was used to make three scratches in the bottom of each well at roughly equal distance from each scratch. Exfoliated cells were washed with PBS for three times. Scratches were imaged under a microscope to define as the control condition after washing the exfoliated cells thoroughly. Then, the cells were treated with different concentrations of Amonifade (0, 2.5, 5 μM) for 24 h. Scratches were observed and imaged using microscope and the migration distance was calculated by using Image J software.

### Transwell invasion assay

Transwell chambers (8-μm micropores, 6.5 mm diameter, Corning Costar, USA) were placed above 24-well plates for invasion assay. Matrigel was dissolved at 4°C overnight and diluted to 1 mg/ml in the medium. 50 μl Matrigel (1 mg/ml, Corning, USA) was added into the upper chambers. After the Matrigel solidified, cells were suspended in medium containing Amonifade (0, 2.5, 5 μM) and seeded at a density of 1×105 cells per well in the upper chambers, and 600 μl medium containing 20% FBS was added to the lower chambers. The upper chambers were placed into the lower chambers, and all systems were incubated at 37°C for 24 h. Next, the upper chambers were rinsed with PBS thrice and fixed in 4% paraformaldehyde for 15 min. Superfluous cells and Matrigel on the upper chambers above the membrane were wiped off. Then, 0.1% crystal violet was used to stain the penetrated cells for 30 min. The migratory cells were captured under a microscope and counted by ImageJ software.

### Western blot analysis

Western blotting was performed as previously described (20), and proteins from cells or tumour tissues were extracted with RIPA lysis buffer containing PMSF or protease inhibitor cocktail (Beyotime, Shanghai, China). The concentration of protein was determined by a BCA protein assay kit (Beyotime, Shanghai, China), and proteins were denatured before storage at -20°C. In brief, the samples were separated by 10% SDS−PAGE and transferred to PVDF membranes. Then, the membranes were sealed with QuickBlock™ Western blocking buffer (Beyotime, Shanghai, China) for 1 h. After rinsing with tris buffered saline with Tween-20 (TBST) three times, the membranes were incubated with primary antibodies overnight at 4°C. The next day, the membranes were rinsed with TBST and incubated with secondary antibodies at room temperature for 1 h. For detection, the membranes were soaked in enhanced ECL solution (Beyotime, Shanghai, China), and images were captured using a UVP BioSpectrum Imaging System (BioSpectrum 510, USA).

### Statistical analysis

In this study, all statistical analyses were performed using Perl software and the R. The Wilcoxon rank sum test was used to compare the two paired groups. p-values < 0.05 were considered statistically significant. All data were expressed as mean ± standard deviation (SD) and analysed using GraphPad Prism software (version 8.0). Statistical analysis was performed by one-way ANOVA or Dunnett’s *post hoc* test. Statistical significance was shown as P<0.05.

## Results

### Research course

**Figure S1** shows the general process of the study: firstly, the UCSC database was used to screen the glioam DEGS, and a total of 809 mitochondrial genes associated with brain tissue were retrieved from the mitochondrial-associated gene dataset, and the differential genes were taken with the mitochondrial genes, and the biology of the intersecting differential genes leading to the disease and the possible mechanisms leading to the disease were explored by Gene Ontology (GO) and Kyoto Encyclopedia of Genes and Genomes (KEGG). Lasso regression analysis was then used to find mitochondrial model genes and the model was validated using CGGA, kamoun and gravendeel datasets. The final analysis was performed using single gene GSEA pathway analysis. Correlations between individual model genes and immune cells were then explored, and the CellMiner database was used to predict the drug sensitivity of the final model markers screened for possible drugs to treat gliomas, and molecular docking was used to explore the mechanisms of their interactions. Ultimately, the proliferation, migration and invasive ability of predicted drugs on cells was explored through tissue samples and U251 and A172 cell lines.

### Identification of differentially expressed DEGs

**Figure 1:**
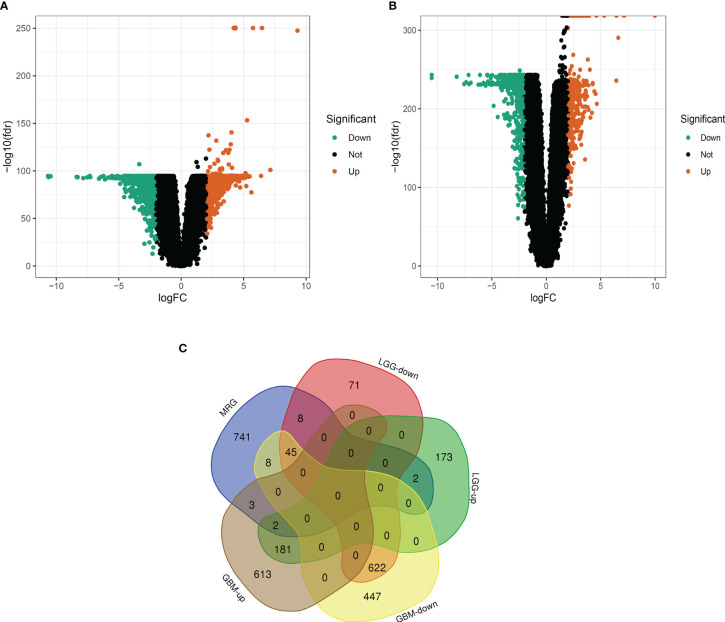
Differential analysis of glioma mitochondria-related genes. **(A)** Volcano map of differentially expressed genes in glioblastoma. Figure **(B)** Volcano map of differentially expressed genes in low-grade gliomas. Figure **(C)** Venn diagram of mitochondria-associated differentially expressed genes in glioblastoma and low-grade glioma.

As shown in **Figure 1**. brain and spinal cord tissue mitochondria-related genes were collated from the MitoCarta 3.0 database of 809. We then matched the 809 MRGs with LGG, GBM-associated differentially expressed mRNA data, and **Figure 1A** shows a volcano map of DEGs in GBM. **Figure 1B** shows the volcano map of DEGs in LGG. The results in **Figure 1C** are:Venn diagram of GBM and LGG mitochondria-associated differentially expressed genes. Forty-seven DEGs were identified, including 45 down-regulated DEGs and 2 down-regulated DEGs shared by LGG and GBM. (|logFC|>2.0, fdrP value <0.05)

### GO and KEGG enrichment analysis of Mit-DEGs

**Figure 2:**
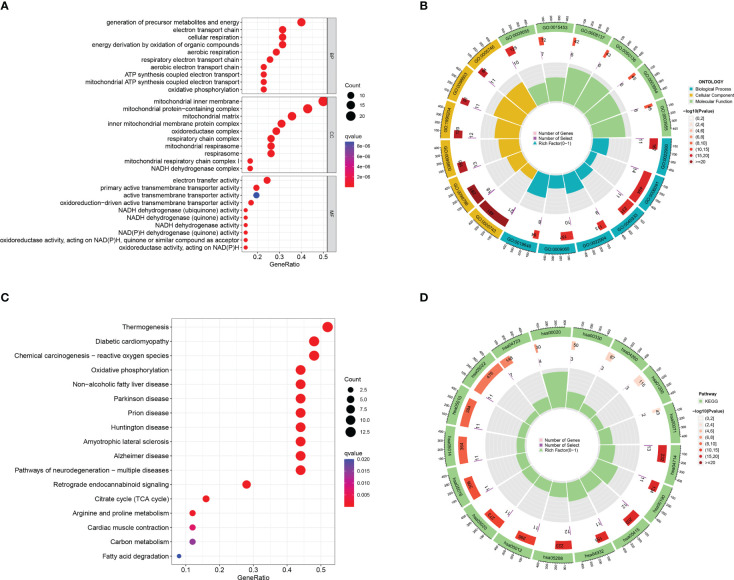
GO and KEGG enrichment analysis of mitochondria-associated differentially expressed genes. **(A)** bubble plot of GO enrichment analysis of 47 mitochondria-associated differentially expressed genes; **(B)** loop of GO enrichment analysis; **(C)** bubble plot of KEGG enrichment analysis of 47 mitochondria-associated differentially expressed genes; **(D)** loop of KEGG enrichment analysis.

Forty-seven DEGs were subjected to GO and KEGG enrichment analyses, with GO analysis mainly enriching for mitochondrial respiratory chain complex assembly, oxidative phosphorylation, mitochondrial translation (**Figures 2A, B**), KEGG analysis was mainly enriched in the Oxidative phosphorylation pathway (**Figures 2C, D**).

### Protein-protein interaction network of Mit-DEGs

The results of the protein-protein interaction network analysis (**Figures S2A, B**) showed that the proteins encoded by the top 10 hub genes were cytochrome c oxidase subunit 4 isoform 1 (*COX4I1*), succinate dehydrogenase cytochrome b560 subunit (*SDHC*), NADH dehydrogenase [ubiquinone] 1β subcomplex subunit 8 (*NDUFB8*), cytochrome c oxidase sub unit 6C (*COX6C*), cytochrome b-c1 complex subunit 7 (*UQCRB*), NADH dehydrogenase [ubiquinone] 1α subcomplex subunit 13 (*NDUFA13*), NADH dehydrogenase [ubiquinone] 1α subcomplex subunit 7 (*NDUFA7*), NADH dehydrogenase [ubiquinone] flavoprotein 2 (*NDUFV2*), NADH dehydrogenase [ubiquinone]1α subcomplex subunit 11 (*NDUFA11*), mitochondrial contact point and cristae organizing system subunit 10 (*MINOS1*).

### Mit-DEGs prognostic model construction

**Figure 3:**
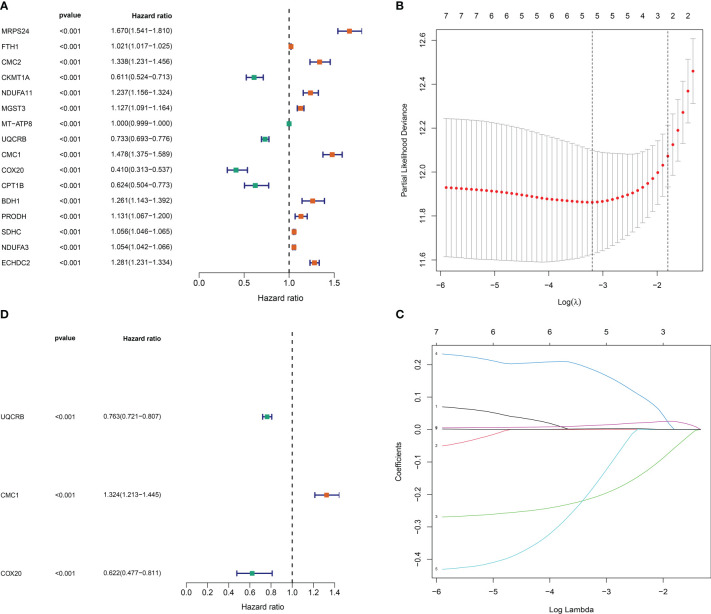
Mitochondrial-associated differential gene prognostic model construction. **(A)** One-way Cox risk regression for mitochondria-associated differential genes; **(B)** vertical coordinate is Binomial Deviance (dichotomous abnormality). **(C)** Each curve in the figure represents the trajectory of each independent variable coefficient. The vertical coordinate is the value of the coefficient, the lower horizontal coordinate is log(λ), and the upper horizontal coordinate is the number of non-zero coefficients in the model at this point. **(D)** Mitochondria-associated differential genetic multifactorial Cox risk regression.

we identified 16 significantly associated DEGs (P<0.001); (**Figure 3A**) of which 11 genes, including Mitochondrial ribosomal protein S24 (*MRPS24*), Ferritin heavy chain (*FTH1*), *CMC2*, *NDUFA11*, Microsomal glutathione S-transferase 3 (*MGST3*), *CMC1*, D-beta-hydroxybutyrate dehydrogenase (*BDH1*), Proline dehydrogenase 1 (*PRODH*), *SDHC*, NADH dehydrogenase [ubiquinone] 1 alpha subcomplex subunit 3 (*NDUFA3*), Enoyl-CoA hydratase domain-containing protein 2 (*ECHDC2*), were identified as risk factors (P< 0.001). While five, including *COX20*, Carnitine O-palmitoyltransferase 1 (*CPT1B*), Mitochondrially encoded atp synthase membrane subunit 8 (*MT-ATP8*), *UQCRB* and Creatine kinase (*CKMT1A*), were considered as protective factors (P< 0.001). To prevent overfitting and eliminate the highly correlated **Mit-**DEGs lasso regression analysis was performed (**Figures 3B, C**). Three DEGs were identified by multifactorial Cox regression analysis, namely: *UQCRB*, *CMC1*, *COX20* and used to tap into mitochondria-related prognostic features. A signature was created to assess the prognostic risk score for each glioma patient = (-0.27065 x *UQCRB* expression) + (0.28075 x *CMC1* expression) + (-0.475444 x *COX20* expression), and the results are shown in (**Figure 3D**): HR for *UQCRB* = 0.763 (0.721-0.807) HR=1.324 (1.213-1.445) for *CMC1* and 0.622 (0.477-0.811) for *COX20*. p<0.001.

### Development of Mit-DEGs related features

**Figure 4:**
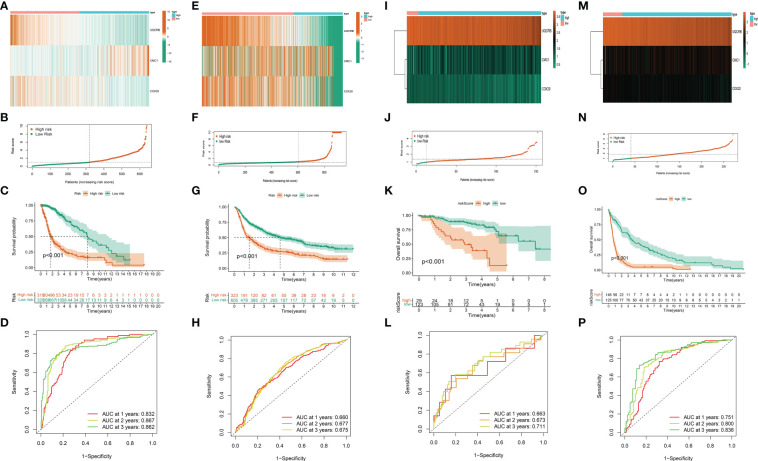
Validation of the mitochondria-associated differential gene prognostic model. **(A)** Expression of UQCRB, CMC1, and COX20 in the TCGA training set; **(B)** Scatter plot of the model in the TCGA training set dividing the samples into high-risk and low-risk groups; **(C)** Distribution of survival status of all samples in the TCGA training set; **(D)** ROC curves of the model at 1, 2, and 3 years in the TCGA training set; **(E–H)** Verification of CGGA validation set; **(I–L)** Verification of kamoun validation set; **(M–P)**Verification of gravendeel validation set.

The risk scores of the 693 glioma patients were calculated as described above, and the patients were divided into high and low risk groups according to the median risk score. *CMC1* was highly expressed in the high-risk group, while *UQCRB* and *COX20* were highly expressed in the low-risk group (**Figure 4A**). The scatter plot of the model in the TCGA training set divided the samples into high-risk and low-risk groups (**Figure 4B**); the survival status distribution of all samples in the TCGA training set (**Figure 4C**); the 1-year, 2-year and 3-year ROC curves of the model in the TCGA training set The ROC curves for the TCGA training set model yielded acceptable AUC values of 0.832, 0.867 and 0.862 for 1, 2 and 3 year survival, respectively (**Figure 4D**).

While the results of our CGGA validation dataset remain consistent (**Figures 4E–H**),ROC curve analysis of the CGGA validation set model yielded acceptable AUC values of 0.660, 0.677, and 0.675 for 1, 2, and 3 year survival, respectively.

The same method was used above to calculate and visualise the results for the kamoun dataset, see (**Figures 4I–L**); while the gravendeel database results are shown in (**Figures 4M–P**).These show similar trends to those observed in the TCGA training set and the validation sets of CGGA, kamoun, and gravendeel. In conclusion, the three mitochondria-related signatures by *UQCRB*, *CMC1*, and *COX20* have the ability to predict OS in gliomas.

### Relationship between risk scoring and clinicopathological factors

**Figure 5:**
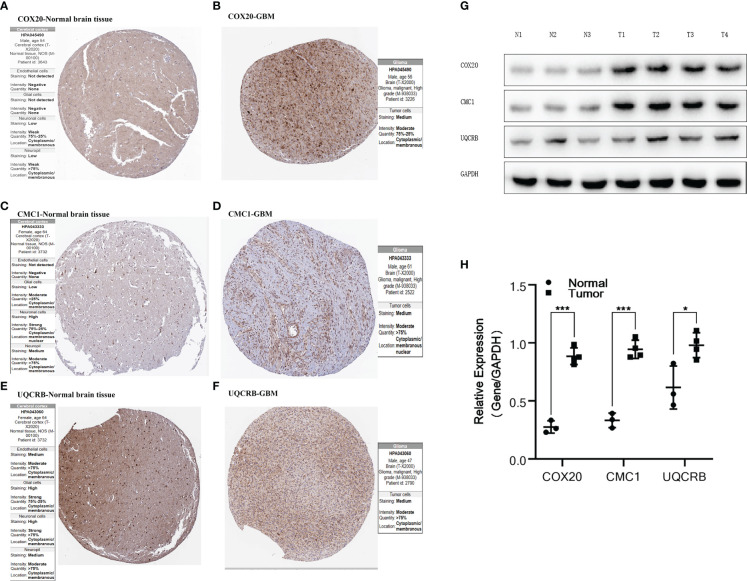
Validation of protein expression by HPA database and Western Blot.**(A, B)** Expression of COX20 in normal brain tissue and glioma; **(C, D)** Expression of CMC1 in normal brain tissue and glioma; **(E, F)** Expression of UQCRB in normal brain tissue and glioma. **(G, H)** Expression of COX20, CMC1 and UQCRB in three normal tissues and four tumor tissues and differential analysis. P values (* < 0.05; *** < 0.001).

**Figure 6:**
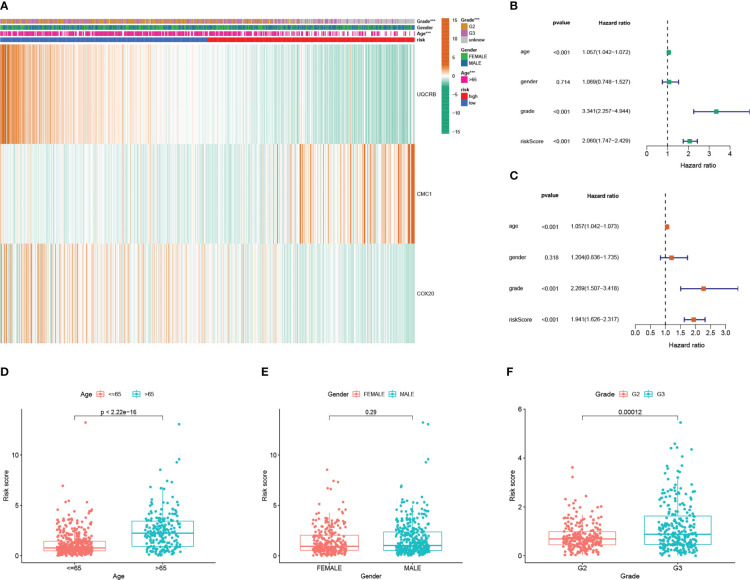
Mitochondrial-associated differential gene prognostic model in clinicopathological performance and independent prognostic analysis **(A)** Heat map of prognostic model and clinicopathological performance in TCGA database; **(B)** TCGA univariate Cox analysis and independent prognostic analysis of clinicopathology; **(C)** multifactorial Cox regression analysis and independent prognostic analysis of clinicopathology; **(D)** differential relationship between age clinical traits in risk group; **(E)** differential relationship between sex clinical traits in risk group; **(F)** differential relationship between staged clinical traits in risk group.

In addition, **Figure 5** shows the expression of 3 key genes and clinicopathological factors in the TCGA database in the high risk group versus the low risk group. The results of the TCGA database, (**Figure 6A**), showed that as the risk score decreased *UQCRB*, *CMC1* gradually increased and *COX20* gradually decreased. Significant differences in pathological grading and age were found between the high- and low-risk groups. (**Figures 6B, C**) TCGA univariate and Multi-factor Cox analysis showed that: Grade, Age (p<0.001), could be used as independent prognostic factors. (**Figures 6D–F**) as: Age, gender, and Grade differed from the model, with Age > 65 years having a higher risk of developing the disease and a worse prognosis (p < 2.22e-16) and G3 having a worse prognosis than G2 stage in Grade (p = 0.00012); while gender made no difference to patient prognosis. Due to the richer clinical information within the CGGA database, the correlation results of analyses performed using the same methodology are shown in **Figure S3**, where (**Figure S3A**) demonstrates the correlation between *UQCRB*, *CMC1* risk and 1p19q_codeletion_status, IDH_mutation_status, Age, Grade, Histology PRS_type was associated with a reduction in risk score, **Figure S3B** shows the univariate independent prognostic analysis for clinical traits: results for: Histology, Grade, Gender,PRS_type,Age,IDH_mutation_status,Chemo_status,1p19q_codeletion_status status, riskScore factor P<0.001. and multifactorial independent prognostic analysis of clinical traits **Figure S3C** suggests: Grade, Age, RS_type, Chemo_status, 1p19q_codeletion_status,IDH_mutation_status, P ≤ 0.05, (**Figures S3D-K**) for Histology, PRS_type,Grade, Gender, Age, IDH_mutation_status, Chemo_status, Radio_status and 1p19q_codeletion_status risk scores, respectively. The risk of age >65 years is higher and the prognosis is worse p=0.0047, the ranking of risk in Grade is: WHO IV > WHO III > WHO II, G3 has a worse prognosis than G2 (p=0.00012) and the risk of Secondary is higher than Recurrent and Primary in PRS_type; in 1p19q_codeletion_status codeletion_status Non-codel had a higher risk than Codel; for IDH_mutation_status Wildtype had a higher risk than Mutant.

The risk is higher for Wildtype than Mutant. There were significant differences between them all with p-values <0.05.

### Validating the performance of mitochondria-related features

**Figure 7:**
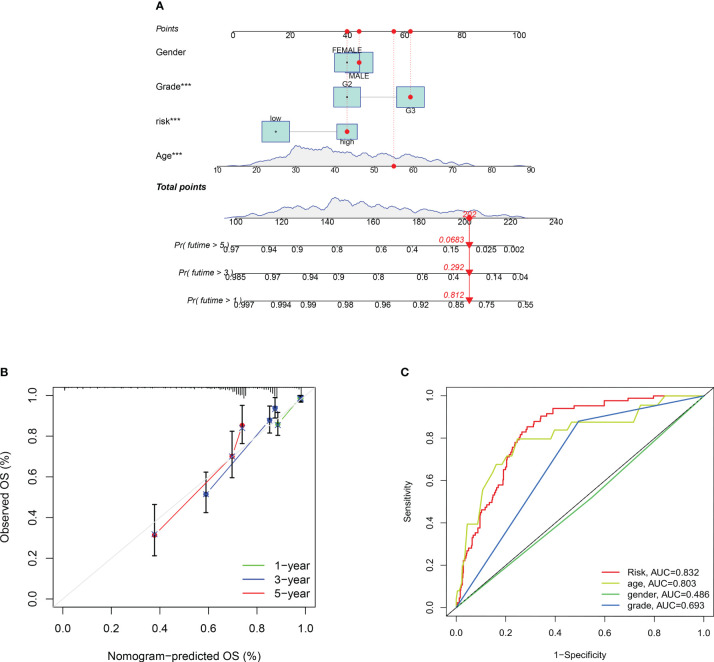
Construction of a nomogram to predict the individualized probability of survival in glioma patients. **(A)** Columnar line plot; **(B)** Columnar line plot calibration curve; **(C)** ROC curve for predicting clinical traits and risk scores in the training set. P values (***P < 0.001).

(**Figure 7A**)Our nomogram are scored according to each prognostic indicator, and by scoring this and adding the scores together to obtain an overall score. (**Figure 7B**) Calibration plots showing that the nomogram graphs are consistent with the ideal model runs. (**Figure 7C**) ROC curves for predicting clinical trait and risk scores for the training set, with AUC values of 0.832 for the risk model, 0.803 for age, 0.486 for gender and 0.693 for staging. In general, AUC values of 0.5 indicate no difference, AUC values of 0.7-0.8 indicate acceptable sensitivity and sensitivity, and AUC values of 0.8-0.9 indicate good sensitivity and specificity, and AUC values above 0.9 indicate excellent sensitivity and specificity. The results of the ROC analysis indicate that the metrics of our nomogram graphs are an acceptable predictive model.

### Mitochondria-related gene signatures are with immune

**Figure 8:**
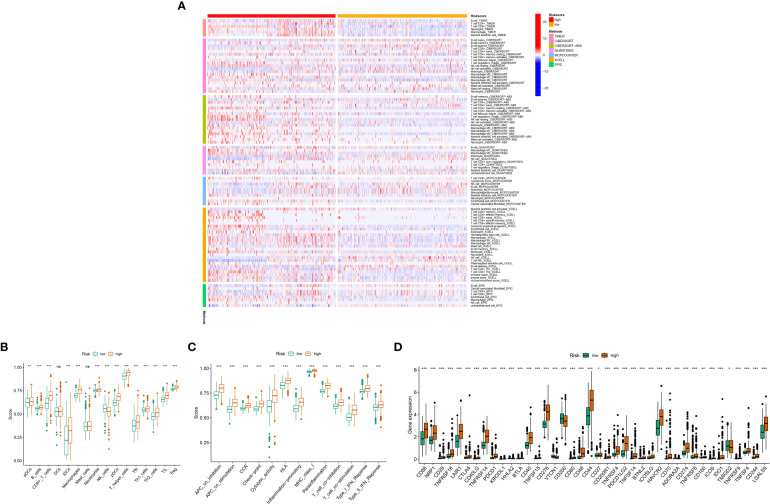
Relationship between mitochondria-associated differential gene prognostic models and immune cell infiltration: **(A)** Heat map of immune responses in high and low risk groups based on CIBERSORT, CIBERSORT-ABS, XCELL, MCPcounter, QUANTISEQ, EPIC and TIMER algorithms. **(B)** High- and low-risk groups with immune cell scores; **(C)** High- and low-risk group populations with immune-related functions. **(D)** Immune checkpoint expression in high and low risk groups. P values (*P < 0.05; **P < 0.01; ***P < 0.001). ns, not statistically significant.

The immune response heat map based on CIBERSORT-ABS, CIBERSORT, XCELL, MCPcounter, QUANT The heat map of the immune response based on seven algorithms, including CIBERSORT-ABS,CIBERSORT, XCELL, QUANT, MCPcounter, EPIC,ISEQ and TIMER, is shown in **Figure 8A**. three mitochondrial models to reflect the state of the immune microenvironment in glioma based on the TIMER algorithm using 22 immune cell types (**Figure 8B**). Unsurprisingly, the content of the immune response scored higher in the high-risk group, and the immune cells that were highly expressed in the high-risk group compared to the low-risk group were: Macrophages, aDCs, CD8+_T_cells, B_cells,iDCs, Th1_ cells, pDCs, Neutrophils,T_helper_cells, Tfh, Th2_cells, Treg and TIL (P < 0.05). While the immune cells that were highly expressed in the low risk group were: NK_cells (P < 0.05), (**Figure 8C**) displayed the relationship between the model and immune function, where the high and low risk group populations with immune-related functions were MHC_class_I, HLA,Type_I_IFN_Reponse, CCR,Check -point,APC_co_stimulation, Cytolytic_activity, Parainflammation, T_cell_co- inhibition, T_cell_co-stimulation, Inflammation-promoting, APC_co_inhibition and Type_II_IFN_Reponse. while (**Figure 8D**) demonstrates the expression of immune checkpoint related genes with risk models, where high expression in high risk populations are. CD86, NRP1, CD28, TNFRSF18, LAIR1, CTLA4, CD40LG, TNFRSF14, PDCD1, KIR3DL1, HHLA2, BTLA, CD40, TNFSF15, CD276, VTCN1, CD200, CD80, CD48, CD44, CD27 CD200R1, TNFRSF4, PDCD1LG2, TNFSF14, BTNL2, ICOSLG, HAVCR2, CD70, ADORA2A, CD274, TNFRSF8, CD160, ICOS, IDO1, TMIGD2, TNFRSF9, TNFSF4, CD244, LGALS9.

**Figures S4A-C** shows the correlation between *UQCRB*, *CMC1* and *COX20* in LGG and GBM tissues, while **Figures S4D-F** shows the correlation between *UQCRB*, *CMC1* and *COX20* in GBM tissues. It can be seen that *UQCRB* was negatively correlated in Natural killer cells and Type 1 T helper cell, Central memory CD4 T cells, Natural killer T cells, Plasmacytoid dendritic cells, Interestingly, Eosinophil, Type 17 T helper cells, Effector memory CD4 T cells, and Activated CD8 T cells were positively correlated with GBM. *COX20* was positively correlated with Type 2 T helper cell in both GBM and LGG, while *COX20* was positively correlated with Central memory CD4 T cell,Mast cell, Type 1 T helper cell, MDSC in both GBM and LGG. helper cell, Macrophage, Central memory CD4 T cell and Natural killer cell.

Overall, there was an increase in survival in patients at low risk, perhaps due to the activation of Monocytes, Mast cells activated, Eosinophils and NK cells activated. These results suggest that the three gene signature models are intimate related to the immune cells and function of gliomas. This provides some theoretical basis for mitochondrial targeting of gliomas when immune cells and other therapies are used.

These results suggest that the three gene signature models are intimate related to the immune cells and function of gliomas. This provides a theoretical basis for mitochondrial targeting of gliomas when immune cells are involved in treatment.

### Model tumour mutational load (TMB) and stem cells are widely associated

The results showed that the percentage of mutations in many genes was lower in the low risk group compared to the high risk scoring group for PTEN (11%), EGFR (19%) (**Figures S5A, B**). Analysis of survival rates after dividing patients into high and low mutation groups showed a meaningful difference between these two groups (**Figure S5C, D**). And the stem cell index was inversely correlated with the risk score (**Figure S5E**). These results suggest a correlation between mutational load and glioblastoma stem cells (GSCs) and mitochondria.

### Exploring the expression of key DEGs

The expression of *COX20*, *CMC1* and *UQCRB* in normal brain tissue and glioma proteins was analysed using the HPA database **Figures 5A–F**. The immunohistochemistry of *UQCRB* protein in normal brain tissue is shown. **Figures 5G-H** shows that the levels of *COX20*, *CMC1* and *UQCRB* are lower in normal brain tissue than in glioma tissue. These results confirm that these three proteins are highly expressed in glioma tissues. This is in general agreement with the results of the HPA database.

### Drug sensitivity analysis and molecular docking

**Figure 9:**
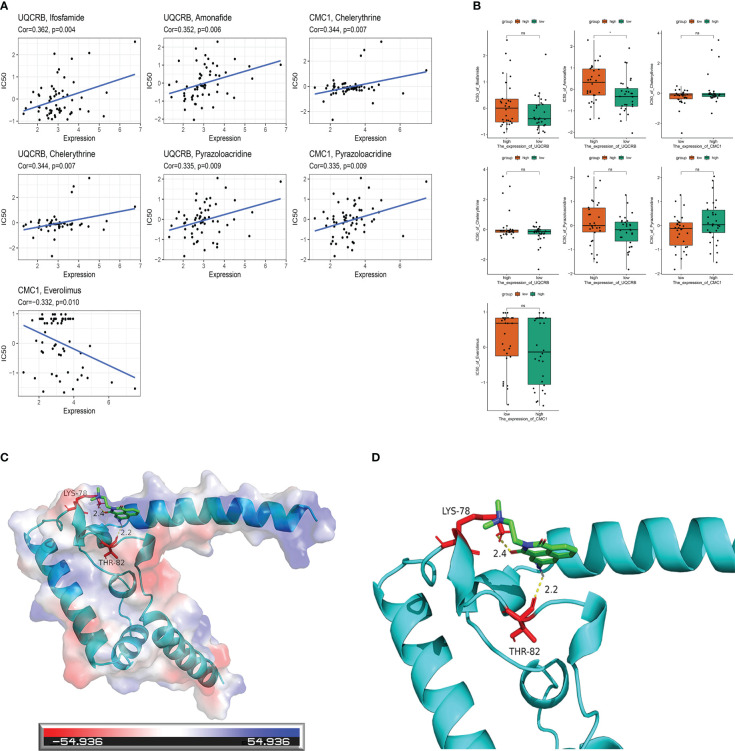
Mitochondria-related prognostic genes and drug sensitivity analysis with molecular docking. **(A)** Correlation analysis of UQCRB, CMC1 corresponding to drug sensitivity in the CellMiner database; **(B)** Correlation analysis of UQCRB, CMC1 expression and corresponding drug sensitivity in the CellMiner database **(C)** UQCRB and Amonafide molecular docking in general; **(D)** UQCRB and Amonafide molecular docking local amplification details.

A drug sensitivity analysis was used to develop a therapeutic agent suitable for all glioma patients. Drug sensitivity results were *UQCRB* positively correlated with Ifosfamide, Amonafide, Chelerythrine and Pyrazoloacridine, *CMC1* positively correlated with Chelerythrine and Pyrazoloacridine, and *CMC1* negatively correlated with Everolimus (**Figure 9A**).Correlation analysis of *UQCRB*, *CMC1* expression and corresponding drug sensitivity showed that only Amonafide sensitivity differed from *UQCRB* expression, p < 0.05 (**Figure 9B**).The docking of *UQCRB* to the Amonafide molecule was carried out showing that the protein is able to bind through residues at a static potential energy of around 54.936 (**Figure 9C**); details of the local amplification of the docking of the *UQCRB* to the Amonafide molecule show that Amonafide is covalently bonded to *UQCRB* through LYS (lysine) THR (threonine) at position 78 (**Figure 9D**).

### Amonifade inhibits the proliferation of SVGp12, U251 and A172 cells

**Figure 10:**
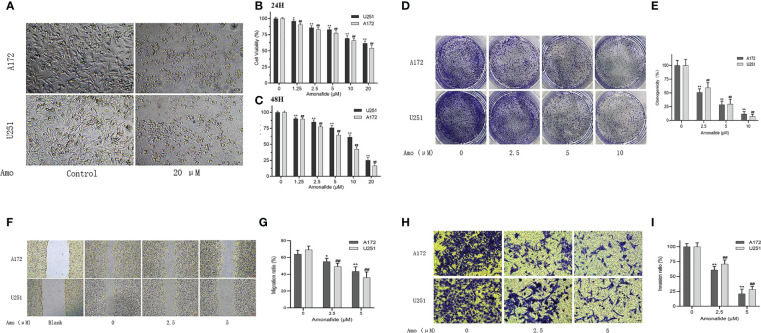
Amonifade inhibits the proliferation, invasion and migration of U251 and A172 cells. **(A)** Effect of Amonifade at concentrations of 0 μM and 20 μM on proliferating cell viability of U251 and A172 cells; **(B, C)** Effect of Amonifade at concentrations of 0 μM, 1.25 μM, 2.5 μM, 5 μM, 10 μM and 20 μM on proliferating cell viability of U251 and A172 cells after 24 and 48 hours of incubation; **(D, E)** Effect of different concentrations of Amonifade on the clonogenic ability of U251, A172 cells after 24 h of treatment; **(F, G)** Effect of different concentrations of Amonifade on the healing ability of U251, A172 cells after 24 h of treatment. **(H, I)** Effect of different concentrations of Amonifade treatment for 24 h on the mailbox of U251, A172 migration invasion. The * symbol means P values < 0.05, the ** symbol means P values < 0.01, and the ## symbol means P values <0.01.

To confirm the inhibited effect of Amonifade on glioma cells, CCK-8 assay and Colony formation assay were used to detect the proliferation of U251 and A172 cells. The results of CCK-8 assay suggested that Amonifade significantly reduced the viability of U251 and A172 cells in a dose- and time-dependent manner (**Figures 10A–C**). Then, the results of colony formation assay showed that the clonogenicity of glioma cells decreased with increasing concentrations of Amonifade. Compared with the control groups, the clonogenicity rates of U251 and A172 cells exposed to 5 µM and 10 µM Amonifade were significantly decreased (**Figures 10D, E**). As shown in (**Figures 10F, G**), compared with the control group, 20 μM Amonifade-treated cells (24 h) showed flattened and loosened in morphology, and most cells gradually break down or die.

Next examined the effect of amo on SVGp12 cells by CCK-8 assay, and found that amofide had an inhibitory effect on SVGp12 cells at concentrations above 5 μM (p < 0.01) (**Figure 6S**), indicating that amo has a certain toxic effect on normal astrocytes. after 10 μM amo intervention for 24 h and 48 h, the SVGp12 The survival rate was 88.7% and 82.3%, respectively, and most of the SVGp12 cells survived (>80%) when amo had a strong inhibitory effect on glioma cells, so we concluded that the toxic effect of 10 μM amo on normal astrocytes was within an acceptable concentration range.

### Amonifade suppresses migration and invasion of U251 and A172 cells

To avoid the inhibitory effects of high concentrations of Amonifade on the proliferation of glioma cells, we chose the concentrations of 2.5 μM and 5 μM Amonifade (the cell viability was over 75% for 24 h) to detect the migratory and invasive abilities of glioma cells. These results in (**Figures 10H, I**) showed that 2.5 μM and 5 μM Amonifade suppressed the migration and invasion of glioma cells significantly compared with the control group.

## Discussion

In this study, we conducted a systematic analysis of the expression and prognosis of mitochondria-associated genes in brain tissue in relation to glioma, and identified three mitochondria-associated genes to construct a novel prognostic model for glioma. The CGGA dataset, kamoun dataset and gravendeel dataset were used to validate the model. The characteristic prognostic nomogram constructed from mitochondria-related genes can be used in the management of glioma patients to guide decisions on the choice of treatment options. Furthermore, mitochondrial features have relatively better sensitivity and specificity as independent prognostic predictors compared to traditional clinicopathological features. We also examined the immune profile between risk groups and the correlation between the corresponding three genes and immune cells in GBM and LGG, respectively, and found that mitochondria-related genes were closely associated with tumour-infiltrating immune cells such as aDCs, B_cells and CD8+_T_cells in gliomas. The higher expression of immune checkpoints in the high-risk group compared to the low-risk group may provide new insights into the role of mitochondria in the immunotherapy of glioma. While there is a wealth of research on the metabolic aspects of tumours, mitochondria are less commonly used as a starting point to study tumour prognosis-related models, and they may still be used as novel biomarkers for glioma.

In recent years, mitochondria have played an important role in the development of cancer. For example, cancer cells lacking mitochondrial DNA (mtDNA) lose their tumourigenic potential unless the tumour cells produce OXPHOS through mitochondria acquired from the host stroma (21). Furthermore, the metabolic activity of mitochondria and their associated ROS production is greatly increased in tumour cells, which require more glucose for energy supply and exhibit higher anabolic activity than normal cells(22). In addition, the metabolic conversion of aerobic glycolysis also promotes cancer cell proliferation by inducing hypoxic activation(23). Toadstatin induces mitochondrial surface membrane association protein A2 and DRP1 zwitterionization, disrupting mitochondrial division/fusion homeostasis and inducing apoptosis in U251 cells (24). One of the most characteristic features of cancer cells is their ability to evade apoptosis, and pro-oncogenic alterations in the MEK/ERK signalling pathway lead to Mfn-1 phosphorylation, thereby preventing apoptosis (25, 26). These results suggest that mitochondria play an important role in many tumours and may be involved in the development and progression of gliomas. However, the specific functions and molecular mechanisms of these mitochondria-related genes require further experimental studies.

On the other hand, activation of immunity is an important mechanism in the fight against cancer growth, and mitochondrial dynamics can have an impact on cancer growth through immune system activity, for example by linking to new evidence on T cells (27). Whereas our immune infiltration and immune cell correlation analysis showed that T cells such as CD8+_T_cells, Th1_cells and Th2_cells are highly correlated among high risk. A new peptide, IFN-γ ELISPOT assays showed that FMACSPVAL efficiently induced sox11-specific CD8 T cells (28). Thus, this novel antigenic peptide epitope appears to be promising as a target for T cell-based immunotherapy in GBM. In contrast, another study has shown that M1 macrophages may induce tumourigenesis by altering the microenvironment, in the same way as the Warburg effect in tumour cells, (29). Macrophage infiltration was significantly increased in the high-risk group in this study, which may have contributed to the poor prognosis of the high-risk group. In a study revealing immune cell infiltration and immunotherapy in low-grade gliomas, high immune infiltration scores were significantly associated with increased levels of CD4 naïve T cell infiltration, and high CD4 naïve T cell expression suggested a good prognosis for patients (30). DEGIn addition, Altered somatic Mitochondrial DNA (mtDNA) or low mtDNA copy number promotes cancer progression and metastasis by activating retrograde mitochondrial signalling (31, 32). In contrast, elimination of mtDNA limits tumourigenesis (33), and in childhood glioblastomas, the average mtDNA content in tumours is significantly lower than in normal brains. mtDNA deletion in pHGG cells promotes cell migration, invasion and therapeutic resistance, and targeted reduction of mtDNA numbers is effective in treating pHGG(34). These results suggest that glioma can activate the immune response of glioma through CD4 T cells,CD8 T cells and macrophages, and regulate the metabolic reprogramming of glioma by reducing mtDNA content and enhancing glycolysis, which may be a new approach for effective treatment of glioma.

Patients treated with radiotherapy have a high recurrence rate due to the radiation resistance of glioma cells. Therapies that target mechanisms of resistance to radiotherapy are urgently needed to improve the radiation response in glioma and thus improve overall patient survival. One study found in radiation-resistant glioma cells that the expression of the NADH ubiquinone oxidoreductase (complex I) subunit was upregulated in mitochondria, where the copy number of mitochondria was increased in radiation-resistant glioma cells, and after treatment of glioma cells with drugs such as mitochondrial complex I inhibitors, drug-resistant glioma cells were found to be resensitised to radiation(35), On the one hand tumour hypoxia and altered metabolic status promote the malignant progression and drug resistance of cancer cells. On the other hand, glioma stem cells are preferentially dependent on mitochondrial metabolism, and therapies targeting mitochondria may be useful for the treatment of glioma stem cells (36). Drug sensitivity analysis showed that Ifosfamide, Amonafide, Chelerythrine and Pyrazoloacridine were positively correlated with mitochondrial gene *UQCRB*. Chelerythrine, Pyrazoloacridine and other drugs were positively correlated with mitochondrial related gene *CMC1*. *CMC1* and *UQCRB* mitochondrial related proteins may improve the radiosensitivity of glioma and reduce the ability of drug resistance, providing new hope for the treatment of recurrent glioma patients. This needs to be further investigated through clinical trials.

Panthenol-cytochrome c reductase-binding protein (*UQCRB*) is the 13.4-kDa subunit of complex III in the mitochondrial respiratory chain, which inhibits hypoxia-induced reactive oxygen species production (37), and mutations in *UQCRB* cause mitochondrial defects and exhibit potent anti-angiogenic activity through reactive oxygen species (ROS)-mediated signaling *in vivo* and *in vitro*(38). On the other hand, downregulation of *UQCRB* expression inhibited the tumour stem cell-like properties of human glioblastoma cells, and synthetic small molecule drugs targeting *UQCRB* significantly inhibited the self-renewal capacity, migration and invasion of U87MG and U373MG-derived glioblastoma stem-like cells (GSCs)(39). These studies suggest that *UQCRB* and its inhibitors may be new therapeutic targets and lead compounds for tumor stem cells in glioblastoma. In turn, *UQCRB* has been studied in tumour prognosis, with both its gene and protein levels in CRC being higher in tumour tissue than in adjacent non-tumour tissue(40). These results suggest that *UQCRB* expression is expected to be a biomarker for tumours. Next, we performed drug sensitivity analysis of this gene and our results showed that *UQCRB* was positively correlated with Amonafide; Amonafide sensitivity differed from *UQCRB* expression, p<0.05. and Amonafide was associated with *UQCRB* through the 78-position LYS (Amonafide is a novel imine derivative with broad preclinical antitumour activity, reaching significant cerebrospinal fluid levels in animal models. A phase II clinical trial was conducted in one study. Of the 22 eligible and evaluable patients treated, tumour regression persisted for more than 1 year in 2 (9%). The remaining patients did not experience tumour regression; one case had stable disease for more than 6 months (41). However, the study used a small study sample and a larger clinical trial should be conducted or could be combined with temozolomide.To demonstrate the anti-glioma activity of Amonafide at the cellular level, we examined the proliferation of U251 and A172 cells by means of a CCK-8 assay and a plate-clone formation assay. The results of the CCK-8 assay showed that Amonifade significantly reduced the viability of U251 and A172 cells in a dose- and time-dependent manner. Scratch healing also decreased with increasing concentrations of Amonifade. Scratches on U251 and A172 cells exposed to 2.5 μM and 5 μM Amonifade were less likely to heal compared to the control group. And 2.5 μM and 5 μM Amonifade inhibited the migration and invasion of glioma cells. These studies suggest that this Amonifade is expected to be a clinical drug for glioma treatment, providing some new treatment options and new individualized treatment modalities for glioma treatment.

However, the study has several shortcomings. Firstly, The data in this study were obtained from a database and the results should be further verified in the clinic to assess the reliability of the predictions of mitochondria-related genes in the clinical setting. Secondly, the effect of mitochondrial changes on glioma immunophenotype has not been studied, which is also our next focus to confirm the role of mitochondrial related genes in glioma immune escape mechanism. In addition, the sensitivity drugs suggested in this study need to be validated in laboratory and clinical trials.

## Conclusion

This study explored the molecular characteristics of mitochondria in gliomas and their prognostic potential(The above includes all grades of LGG and GBM), revealing three mitochondrial prognostic-related biological functions and key signalling pathways that will contribute to further understanding of the molecular mechanisms underlying glioma development. This study develops a new prognostic signature as well as screens for personalised therapeutic agents. This will enrich the diagnostic and therapeutic strategies for glioma patients, thus providing a new strategy for targeting mitochondria and immunotherapy.

## Data availability statement

The original contributions presented in the study are included in the article/**Supplementary Material**. Further inquiries can be directed to the corresponding authors.

## Ethics statement

The studies involving human participants were reviewed and approved by Clinical Research Projects under MedicalEthics Committee, Zhongnan Hospital of Wuhan University. Written informed consent for participation was not required for this study in accordance with the national legislation and the institutional requirements.

## Author contributions

JW conceived the project. JW, JZ, YC and CQ designed the study. JW drafted the manuscript. YZ, SC, NX directed the study. JW, JZ, YC and CQ collected the public data. JW, YZ and SC, NX revised the manuscript. All authors contributed to the article and approved the submitted version.
