# Supplementary figures and images for: Novel prognostic features and personalized treatment strategies for mitochondria-related genes in glioma patients
Source: Front Endocrinol (Lausanne). 2023 Apr 5;14:1172182. doi: 10.3389/fendo.2023.1172182 (PMC10113561; doi:10.3389/fendo.2023.1172182)

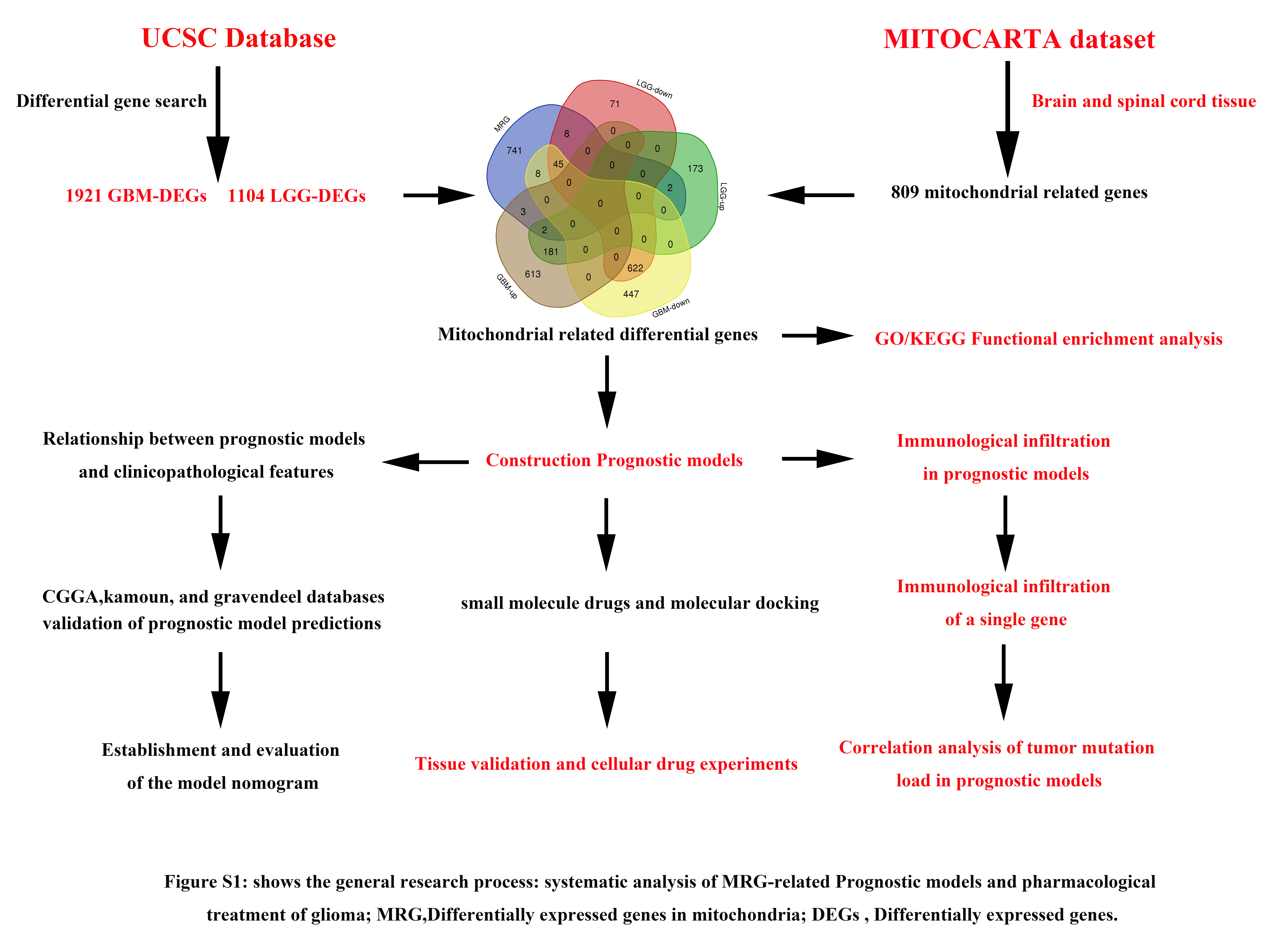

Supplement: Supplementary file 2 [file Image_1.tif]

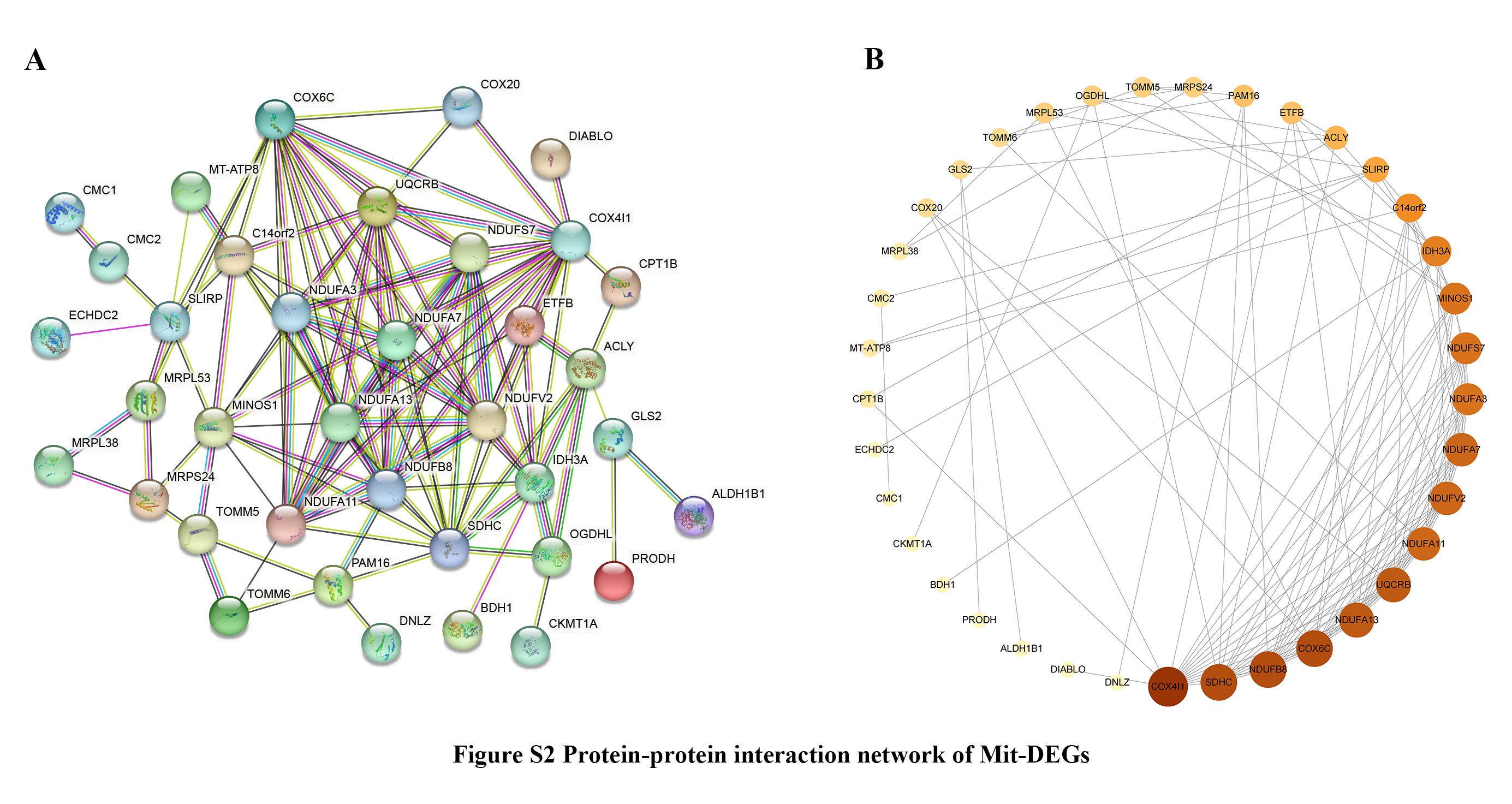

Supplement: Supplementary file 3 [file Image_2.tif]

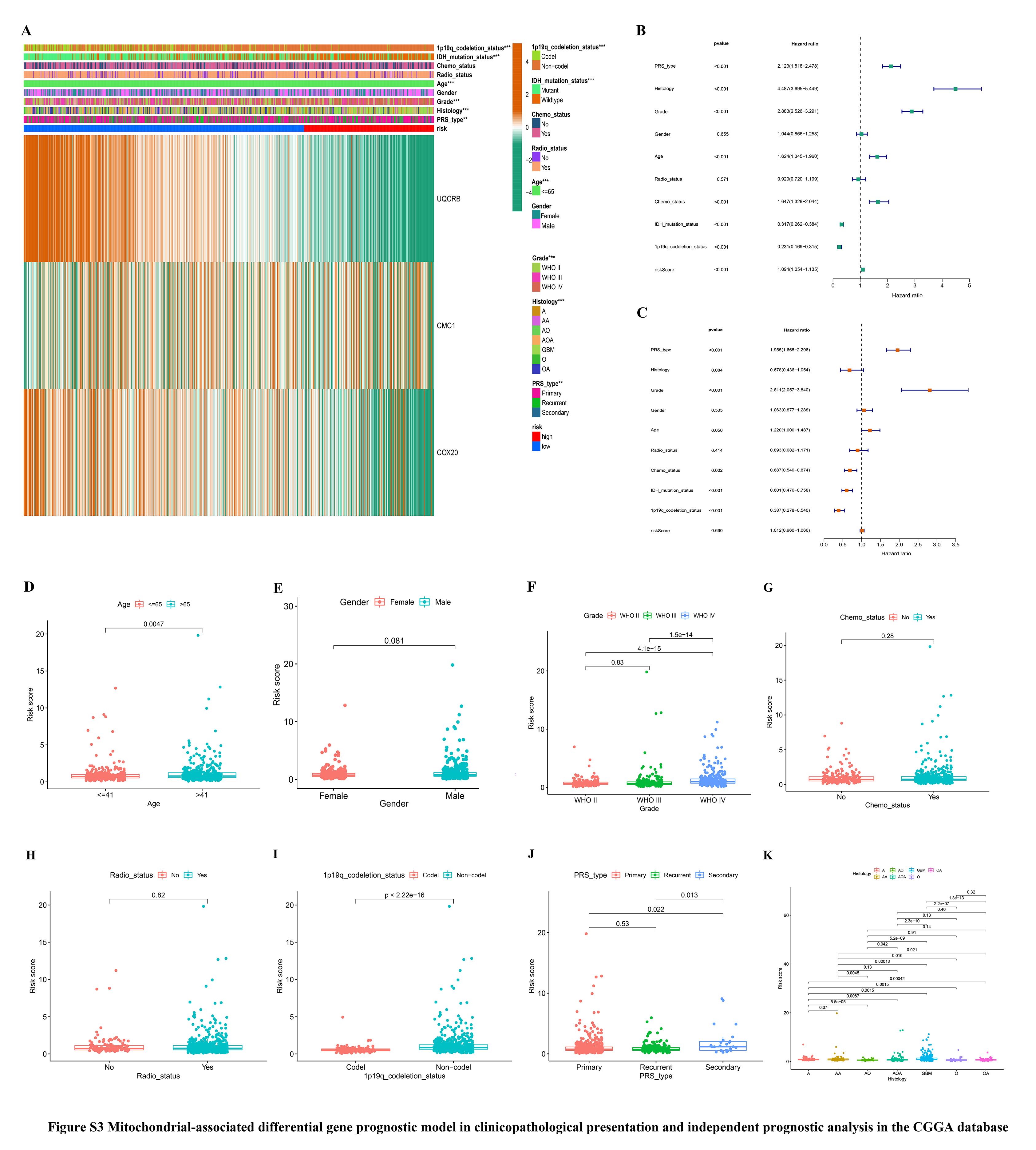

Supplement: Supplementary file 4 [file Image_3.jpeg]

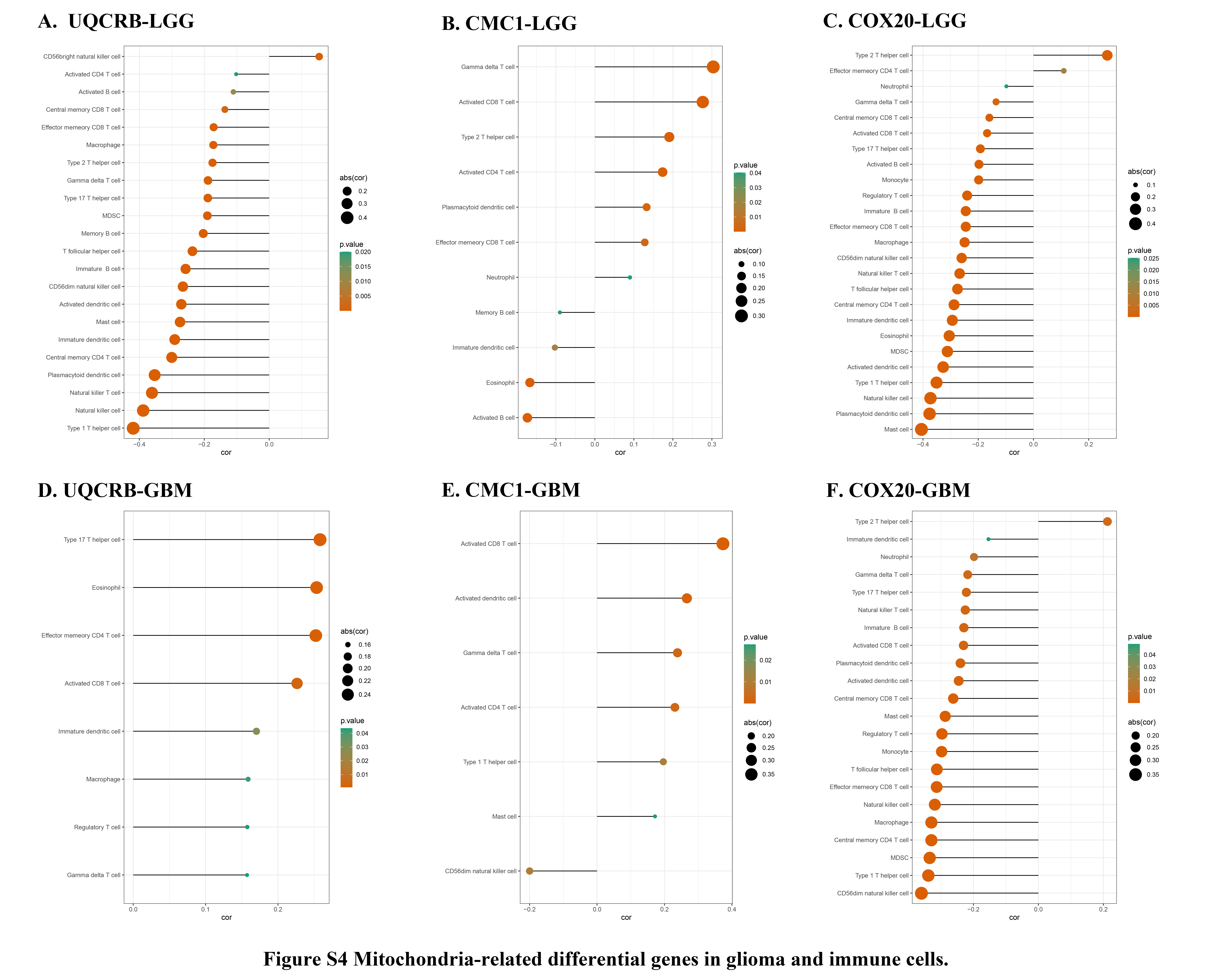

Supplement: Supplementary file 5 [file Image_4.jpeg]

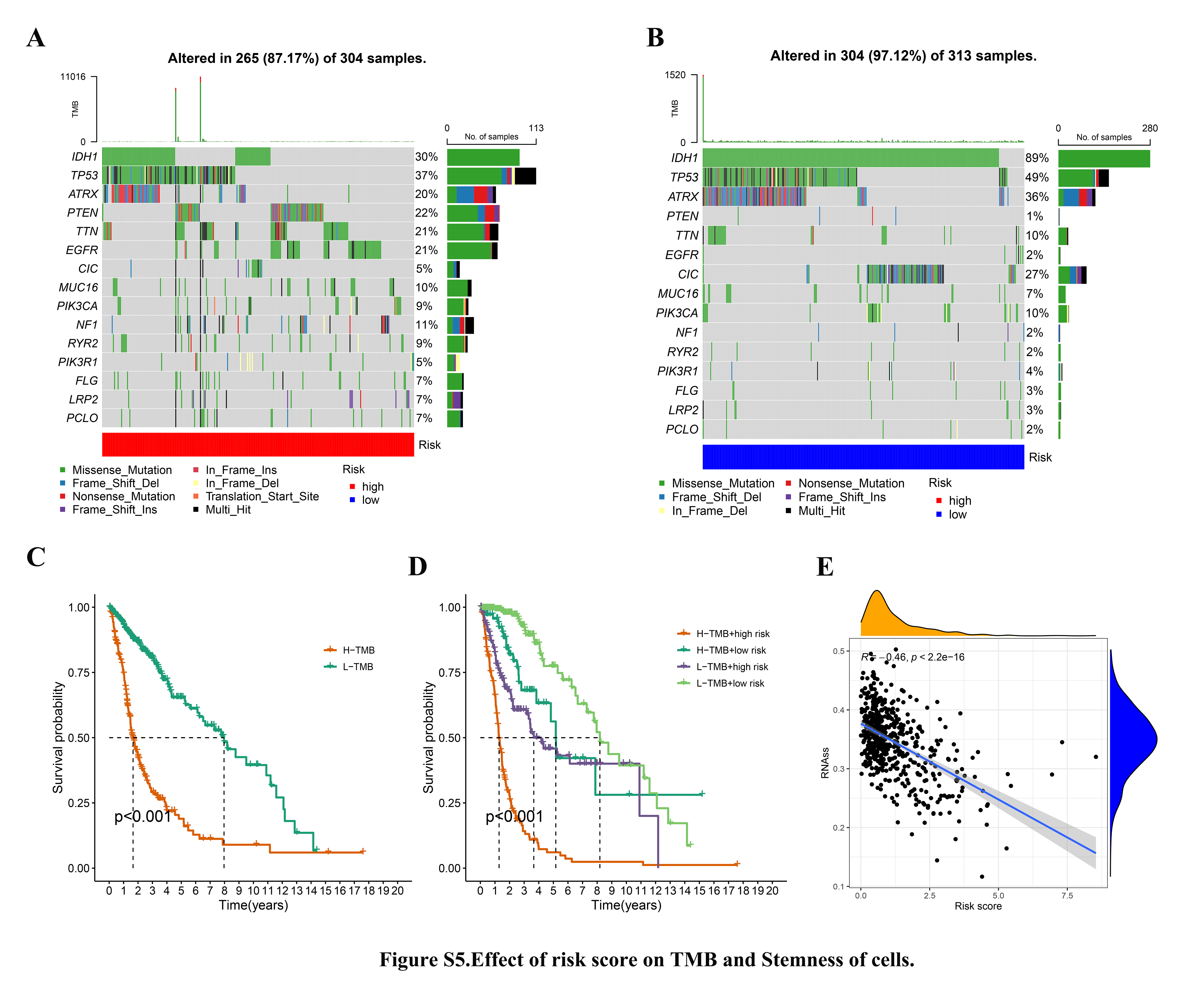

Supplement: Supplementary file 6 [file Image_5.jpeg]

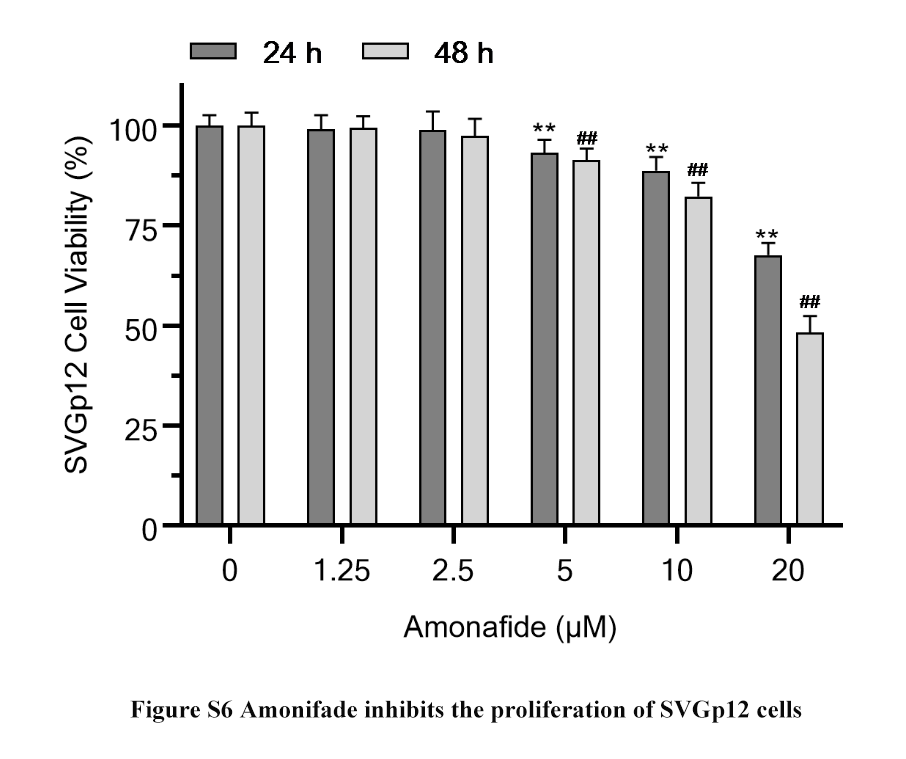

Supplement: Supplementary file 7 [file Image_6.tif]
